# Supplementary material for: Senolytic treatment with fisetin reverses age-related endothelial dysfunction partially mediated by SASP factor CXCL12
Source: bioRxiv. 2025 Aug 18:2025.08.13.670216. Preprint. [Version 1] doi: 10.1101/2025.08.13.670216 (PMC12393244; doi:10.1101/2025.08.13.670216)
Supplement: 1 [file NIHPP2025.08.13.670216v1-supplement-1.pdf]

bioRxiv preprint doi: https://doi.org/10.1101/2025.08.13.670216; this version posted August 18, 2025. The copyright holder for this preprint (which was not certified by peer review) is the author/funder, who has granted bioRxiv a license to display the preprint in perpetuity. It is made available under aCC-BY 4.0 International license.

SUPPLEMENTAL METHODS

Animals and experimental design.

Mouse experiments, including the import, housing, experimental procedures, and euthanasia, were performed strictly under an Animal Study Proposal (ASP #496-LCS-2026) and associated amendments, reviewed and approved by the Animal Care and Use Committee (ACUC) of the National Institute on Aging (NIA). All mice were group-housed at 72°F set point +/-3 under a standard 12 h light/dark cycle and fed ad libitum. Relative humidity was maintained at 30-70%.

For the intervention period, young (6 months) and old (27 months) wildtype mice were randomly assigned to receive vehicle (10% ethanol, 30% PEG400 and 60% Phosal 50 PG) or fisetin (100 mg/kg/day in vehicle). For the young mice, 12 mice (6M/6F) received the vehicle and 12 mice (6M/6F) received fisetin, and for old mice, 8 mice (3M/5F) received vehicle and 10 mice (4M/6F) received fisetin. Treatment was administered via oral gavage using an intermittent dosing paradigm – one week on; two weeks off; one week on, as previously established senolytic dosing paradigm[1,2]. Mice were sacrificed one to two weeks following the final dose to rule out any acute effects of the compound as the terminal half-life of fisetin is ~3.1 hours in plasma[3].

Aortic single-cell RNA sequencing.

Aortic single-cell transcriptomic analysis was performed, as previously described[4]. Briefly, mice were anesthetized with ketamine and xylazine and blood was extracted using cardiac puncture. The whole aorta was collected after left ventricular perfusion with 10 ml of PBS and quickly transferred to cold PBS. To prepare a single-cell suspension, perivascular adipose tissue was removed and whole aortas from three mice (per sex and treatment group) were cut into ~ 1-mm pieces and digested with an enzyme solution consisting of 10 mg/ml Collagenase II (Gibco) and 1 mg/ml elastase (Worthington Biochemical Corp.) in Dulbecco's Modified Eagle Medium (DMEM; 3 ml/sample) for 15 min at 37°C in a tissue culture incubator. The cell suspension was strained through a 40-μm filter and centrifuged at 500 x g for 5 min. Cells were resuspended in DMEM supplemented with 15% fetal bovine serum (FBS) and 5 mM of EDTA and then stained with 2 μg/ml of propidium iodide for 5 min to assess cell viability and single, viable cells were sorted using Fluorescence-Activated Cell Sorting (FACS) Aria FUSION (BD Bioscience) for 10x Genomics library preparation. The single-cell libraries were prepared with Chromium Next GEM Single Cell 3' Kit v3.1 (10x Genomics) with Chromium

bioRxiv preprint doi: https://doi.org/10.1101/2025.08.13.670216; this version posted August 18, 2025. The copyright holder for this preprint (which was not certified by peer review) is the author/funder, who has granted bioRxiv a license to display the preprint in perpetuity. It is made available under aCC-BY 4.0 International license.

Next GEM Chip G Single Cell Kit (10x Genomics) according to manufacturer's protocol with Chromium Controller (10x Genomics). Briefly, ~10,000 single cells were used for GEM generation, and the cDNAs were then checked on the Agilent Bioanalyzer with High Sensitivity DNA kit (Agilent). Prepared cDNAs were then used for library preparation that were checked on the Agilent Bioanalyzer with DNA 1000 kit (Agilent Technologies). The libraries were sequenced with Illumina NovaSeq 6000 sequencer at a depth of 70,000-120,000 reads per cell. Sequencing data are deposited in the NCBI's Gene Expression Omnibus repository GSE296698 (token: yrajmsyujxgjbql).

Single-cell RNA sequencing data were processed using Cell Ranger (version 8.0.1) with the mouse reference reference GRCm39-2024-A (10x Genomics). The obtained read count matrices were subsequently analyzed in R using the Seurat package, version 5.2.0 with default parameters in all functions, unless otherwise specified[5]. Standard quality control filtering was applied to each sample to eliminate low-quality cells and potential doublets from downstream analysis. Filtering also removed cells containing more than 25% mitochondrial RNAs, expressing fewer than 200 or greater than 7,000 transcripts (**Figure S1A** and **S1B**). RNAs that were detected in less than 3 cells were excluded from the analysis. For each sample, we analyzed RNA data with "LogNormalize" method followed by running the FindVariableFeatures function to select 2,000 most variable RNAs for dimensionality reduction. The FindIntegrationAnchors function was applied to choose anchors for data integration. After performing Principal Component Analysis (PCA), the top 40 PCA dimensions were determined by the ElbowPlot method and used to create the Uniform Manifold Approximation and Projection (UMAP) with the resolution parameter set to 0.3. Unsupervised clustering of the merged revealed 29 clusters (**Figure S1C**). Differentially expressed marker RNAs for each cluster were identified with the FindAllMarkers function, and the FindMarkers function was used to find differentially expressed genes between experimental conditions. The main cell types were identified using marker genes of each cluster in combination with data from literature (**Figure S1D** and **S1E**) and revealed 11 distinct cell types in the aorta (**Figure S1F-H**). To identify subclusters within the cell types, the analysis was rerun separately on the cells of each cluster and the UMAPs were created using the resolution 0.3. Senescent cells assessment scoring was performed with Seurat function AddModuleScore. Differentially abundant RNA (DAR) testing was performed with the following cutoffs: adjusted p-value < 0.05, average log2 fold change > 0.25, and minimum percentage

bioRxiv preprint doi: https://doi.org/10.1101/2025.08.13.670216; this version posted August 18, 2025. The copyright holder for this preprint (which was not certified by peer review) is the author/funder, who has granted bioRxiv a license to display the preprint in perpetuity. It is made available under aCC-BY 4.0 International license.

of cells in either group > 0.1. Pathway analysis was performed using KEGG (version 111.0)[6] and Reactome (version 90)[7] and was based on DAR testing.

Plasma protein quantification.

For multiplex analysis, plasma was thawed and centrifuged at 16,000 x g for 4 min. Custom murine Luminex Assay kits were designed by R&D Biosystems to include the following analytes: CCL4, CCL7, CCL8, CXCL12, FGF Basic, GDF15, IGFBP3, PAI-1, S100A9, and TIMP4. Plasma was diluted 1:10 using the Calibrator Diluent RD6-52 provided in the kit. Standards (provided with the kit), blanks, and plasma were incubated with the microparticle cocktail for 2 h at 25°C, followed by incubation with Biotin-Antibody cocktail for 1 h. The final incubation lasted 30 min with Streptavidin-PE and shaking at 25°C prior to running the plate on the Bio-Rad Bioplex-200 Instrument. Each incubation was followed by washing 3 times with Wash Buffer (provided in the kit). Instrument settings were adjusted to the following: 50 µl sample volume, Bio-Plex MagPlex Beads (Magnetic), Double Discriminator Gates set at 8,000 and 23,000, low RP1 target value for the CAL2 setting, 50 count/region. The results were analyzed with the Bio-Plex Manager software.

Plasma-mediated vascular endothelial function.

To assess the role of the circulating SASP milieu (plasma) and CXCL12 on endothelial function, an *ex vivo* isolated artery model was leveraged, as previously described[8]. In brief, carotid arteries were excised from young (3-6 month), intervention-naïve wildtype mice and cannulated onto pressure myographs. Plasma collected from young vehicle, old vehicle and old-fisetin treated mice was diluted in a solution containing 5% sex-matched plasma, 1% penicillin-streptomycin antibiotic cocktail, and 94% modified Krebs buffer corrected to pH 7.3. The diluted plasma samples were perfused luminally through the pressurized arteries for 24 h prior to assessing endothelial function. Following plasma perfusion, endothelial function was measured endothelium-dependent dilation (EDD) and endothelium-independent dilation (EID) in response to increasing doses of acetylcholine (ACh) and sodium nitroprusside (SNP), respectively, as described previously[1,8]. In brief, after vessels were pre-constricted with phenylephrine (PE; 2 mM; Sigma-Aldrich), EDD was assessed by measuring increases in luminal diameter in response to increasing concentrations of ACh (1×10^{-9} to 1×10^{-4} M; Sigma-Aldrich). Following EDD, EID was assessed by measuring the increase in luminal diameter in response to increasing concentrations of SNP, an exogenous NO donor (1×10^{-10} to 1×10^{-4} M; Sigma-Aldrich). All dose-

bioRxiv preprint doi: https://doi.org/10.1101/2025.08.13.670216; this version posted August 18, 2025. The copyright holder for this preprint (which was not certified by peer review) is the author/funder, who has granted bioRxiv a license to display the preprint in perpetuity. It is made available under aCC-BY 4.0 International license.

response data are presented as percent dilation relative to maximum diameter to account for differences in baseline vessel diameter.

Arterial mitochondrial superoxide bioactivity.

Arterial mitochondrial superoxide bioactivity was assessed using the mitochondrial-specific superoxide spin probe 1-hydroxy-4-[2-triphenylphosphonio-acetamido]-2,2,6,6-tetramethylpiperidine (mitoTEMPO-H; Enzo Life Sciences) by electron paramagnetic resonance (EPR) spectrometry, as previously described[1,9]. In short, 1 mm aortic rings were incubated in 5% sex-matched plasma from young, old vehicle- or old fisetin-treated mice in DMEM for 24h. Following plasma exposure, aortic rings were incubated in Krebs/HEPES buffer containing 0.5 mM mitoTEMPO-H at 37°C for 1 h. Samples were analyzed using aMS300 Xband EPR spectrometer (Magnettech).

Cultured endothelial cell nitric oxide (NO) and mitochondrial superoxide bioactivity.

Human aortic endothelial cells (HAECs; PromoCell; used at passage 3-4; female, age: 80 years, non-smoker, free from known CVD) were cultured in a 96-well glass bottom plate (CellVis) under standard culture conditions (37.5°C, 100% relative humidity, 5% CO2). HAECs were grown in basal media (Endothelial Cell Growth Medium-2 [EGM-2] BulletKit; PromoCell) supplemented with 5% plasma collected from young, old vehicle, and old fisetin-treated mice for 2 h. HAECs grown in basal media and 5% FBS were used as a control condition. HAECs were stained with the fluorescent probe Hoechst (nuclei stain; Thermo Fisher) and either 10 μM diaminorhodamine-4M AM (DAR-4M AM; Sigma-Aldrich; to quantify NO production) for 45 min or 5 μM MitoSOX (Thermo Fisher; to quantify mitochondrial superoxide bioactivity) for 30 min. Live HAECs were imaged at 20x using wide-field fluorescence microscopy (EVOS M7000 Imaging System; Thermo Fisher) under standard incubation conditions. HAECs stained with DAR-4M AM were imaged before and 6 min after the addition of 100 μM acetycholine (Sigma) to stimulate NO production[10] and mitochondrial superoxide bioactivity was assessed under basal (unstimulated) conditions[11]. Images were quantified using Celleste 5.0 Image Analysis Software (Thermo Fisher) as previously described[10,11] and normalized to the FBS condition on each plate to control for potential variation between plates.

Senescence-associated β-galactosidase (SA-β-Gal) staining.

Arterial and cultured endothelial cell SA-β-Gal staining was performed using the Senescence Detection Kit (Abcam) according to the manufacturer's instructions. Briefly, 1 mm aortic rings or HAECs (described

bioRxiv preprint doi: https://doi.org/10.1101/2025.08.13.670216; this version posted August 18, 2025. The copyright holder for this preprint (which was not certified by peer review) is the author/funder, who has granted bioRxiv a license to display the preprint in perpetuity. It is made available under aCC-BY 4.0 International license.

above) were incubated in 5% sex-matched plasma from young, old vehicle- or old fisetin-treated mice in DMEM or EGM-2, respectively, for 24 h. Following plasma exposure, aortas and HAECs were washed with PBS, fixed (fixing solution provided in the kit), and incubated with the X-gal solution overnight. Aortic rings were then washed, frozen in OCT compound, and stored at -80°C until the time of sectioning. Aortic samples were later sectioned (7 µm; Leica CM300, Leica Biosystems) and plated in poly-L-lysine coated slides. Aortas and HAECs were stained with DAPI overnight and images were captured using bright-field and fluorescent microscopy (EVOS M7000 Imaging System; Thermo Fisher) at 10X magnification and quantified using ImageJ, as described[12].

Cultured endothelial cell gene expression.

mRNA gene expression was measured in HAECs following incubation with 5% plasma from young, old vehicle or old fisetin-treated mice cultured in EGM-2 for 24 h. Briefly, RNA was extracted using the RNeasy mini kit (Qiagen). cDNA was synthesized using the iScript cDNA synthesis kit (Bio-Rad Laboratories). Transcripts of *Cdkn2a, Cdkn1a, Cdh5, Pecam1, Tgfb1,* and *Acta2* were analyzed using a StepOnePlus Real-Time PCR System (Applied Biosystems) in 96-well plates and the Taqman OpenArray (Applied Biosystems) was used as a master mix, as described. SimpleSeq DNA sequencing (Quintara Biosciences) was used to validate PCR products.

CXCL12 protein addition and inhibition.

Recombinant mouse CXCL12 protein (R&D Systems, Cat. #460-SD-050/CF) in sterile PBS was added back to plasma from young and old fisetin-treated mice, such that the concentration of CXCL12 matched the average levels in old vehicle-treated mice (3210 pg/ml) for CXCL12 add-back experiments. 1 µg/ml of LIT-927 (Selleckchem, Cat. #S8813)[13] in DMSO was added to the old vehicle plasma for CXCL12 inhibition experiments.

bioRxiv preprint doi: https://doi.org/10.1101/2025.08.13.670216; this version posted August 18, 2025. The copyright holder for this preprint (which was not certified by peer review) is the author/funder, who has granted bioRxiv a license to display the preprint in perpetuity. It is made available under aCC-BY 4.0 International license.

SUPPLEMENTAL REFERENCES

1. Mahoney SA, Venkatasubramanian R, Darrah MA, Ludwig KR, VanDongen NS, Greenberg NT, Longtine AG, Hutton DA, Brunt VE, Campisi J, et al. Intermittent supplementation with fisetin improves arterial function in old mice by decreasing cellular senescence. *Aging Cell*. 2023:e14060. doi: 10.1111/acel.14060
2. Yousefzadeh MJ, Zhu Y, McGowan SJ, Angelini L, Fuhrmann-Stroissnigg H, Xu M, Ling YY, Melos KI, Pirtskhalava T, Inman CL, et al. Fisetin is a senotherapeutic that extends health and lifespan. *EBioMedicine*. 2018;36:18-28. doi: 10.1016/j.ebiom.2018.09.015
3. Jo JH, Jo JJ, Lee JM, Lee S. Identification of absolute conversion to geraldol from fisetin and pharmacokinetics in mouse. *J Chromatogr B Analyt Technol Biomed Life Sci*. 2016;1038:95-100. doi: 10.1016/j.jchromb.2016.10.034
4. Mazan-Mamczarz K, Tsitsipatis D, Carr A, Childs B, Rocha Dos Santos C, Anerillas C, Romero B, Gregg J, Michel M, Munk R, et al. Single-cell and spatial transcriptomics uncovers the role of senescent vascular cells in pathological arterial remodeling during atherosclerosis. In: Research Square Platform LLC: Research Square Platform LLC; 2023.
5. Hao Y, Stuart T, Kowalski MH, Choudhary S, Hoffman P, Hartman A, Srivastava A, Molla G, Madad S, Fernandez-Granda C, et al. Dictionary learning for integrative, multimodal and scalable single-cell analysis. *Nat Biotechnol*. 2024;42:293-304. doi: 10.1038/s41587-023-01767-y
6. Kanehisa M, Furumichi M, Sato Y, Matsuura Y, Ishiguro-Watanabe M. KEGG: biological systems database as a model of the real world. *Nucleic Acids Res*. 2025;53:D672-D677. doi: 10.1093/nar/gkae909
7. Milacic M, Beavers D, Conley P, Gong C, Gillespie M, Griss J, Haw R, Jassal B, Matthews L, May B, et al. The Reactome Pathway Knowledgebase 2024. *Nucleic Acids Res*. 2024;52:D672-D678. doi: 10.1093/nar/gkad1025
8. Mahoney SA, VanDongen NS, Greenberg NT, Venkatasubramanian R, Rossman MJ, Widlansky ME, Brunt VE, Bernaldo de Quirós Y, Seals DR, Clayton ZS. Role of the circulating milieu in age-related arterial dysfunction: a novel. *Am J Physiol Heart Circ Physiol*. 2024. doi: 10.1152/ajpheart.00014.2024
9. Clayton ZS, Rossman MJ, Mahoney SA, Venkatasubramanian R, Maurer GS, Hutton DA, VanDongen NS, Greenberg NT, Longtine AG, Ludwig KR, et al. Cellular Senescence Contributes to Large Elastic Artery Stiffening and Endothelial Dysfunction With Aging: Amelioration With Senolytic Treatment. *Hypertension*. 2023;80:2072-2087. doi: 10.1161/HYPERTENSIONAHA.123.21392
10. Murray KO, Ludwig KR, Darvish S, Coppock ME, Seals DR, Rossman MJ. Chronic mitochondria antioxidant treatment in older adults alters the circulating milieu to improve endothelial cell function and mitochondrial oxidative stress. *Am J Physiol Heart Circ Physiol*. 2023;325:H187-H194. doi: 10.1152/ajpheart.00270.2023
11. Darvish S, Murray KO, Ludwig KR, Avalani KH, Craighead DH, Freeberg KA, Bevers S, Reisz JA, D'Alessandro A, Moreau KL, et al. Preservation of Vascular Endothelial Function in Late-Onset Postmenopausal Women. *Circ Res*. 2025;136:455-469. doi: 10.1161/CIRCRESAHA.124.325639
12. Kurz DJ, Decary S, Hong Y, Erusalimsky JD. Senescence-associated (beta)-galactosidase reflects an increase in lysosomal mass during replicative ageing of human endothelial cells. *J Cell Sci*. 2000;113 (Pt 20):3613-3622. doi: 10.1242/jcs.113.20.3613
13. Xiong Q, Zhang N, Zhang M, Wang M, Wang L, Fan Y, Lin CY. Engineer a pre-metastatic niched microenvironment to attract breast cancer cells by utilizing a 3D printed polycaprolactone/nano-hydroxyapatite osteogenic scaffold - An in vitro model system for proof of concept. *J Biomed Mater Res B Appl Biomater*. 2022;110:1604-1614. doi: 10.1002/jbm.b.35021

bioRxiv preprint doi: https://doi.org/10.1101/2025.08.13.670216; this version posted August 18, 2025. The copyright holder for this preprint (which was not certified by peer review) is the author/funder, who has granted bioRxiv a license to display the preprint in perpetuity. It is made available under aCC-BY 4.0 International license.

SUPPLEMENTAL FIGURES

A

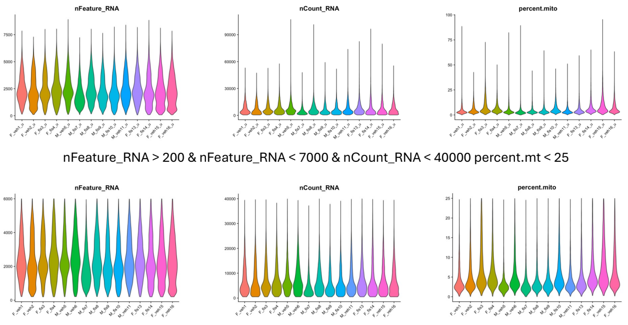

B

	unfiltered	filtered	% retained
F_veh1_Y	8495	8411	99%
F_veh2_Y	8852	8719	98%
F_fis3_Y	7322	6835	93%
F_fis4_Y	7192	7013	98%
M_veh5_Y	7368	7220	98%
M_veh6_Y	6569	6370	97%
M_fis7_Y	14861	14783	99%
M_fis8_Y	8772	8668	99%
M_fis9_O	10068	9969	99%
M_fis10_O	9299	9086	98%
M_veh11_O	8535	8416	99%
F_fis13_O	8341	8138	98%
F_fis14_O	7327	6948	95%
F_veh15_O	6959	6309	91%
F_veh16_O	10591	10197	96%

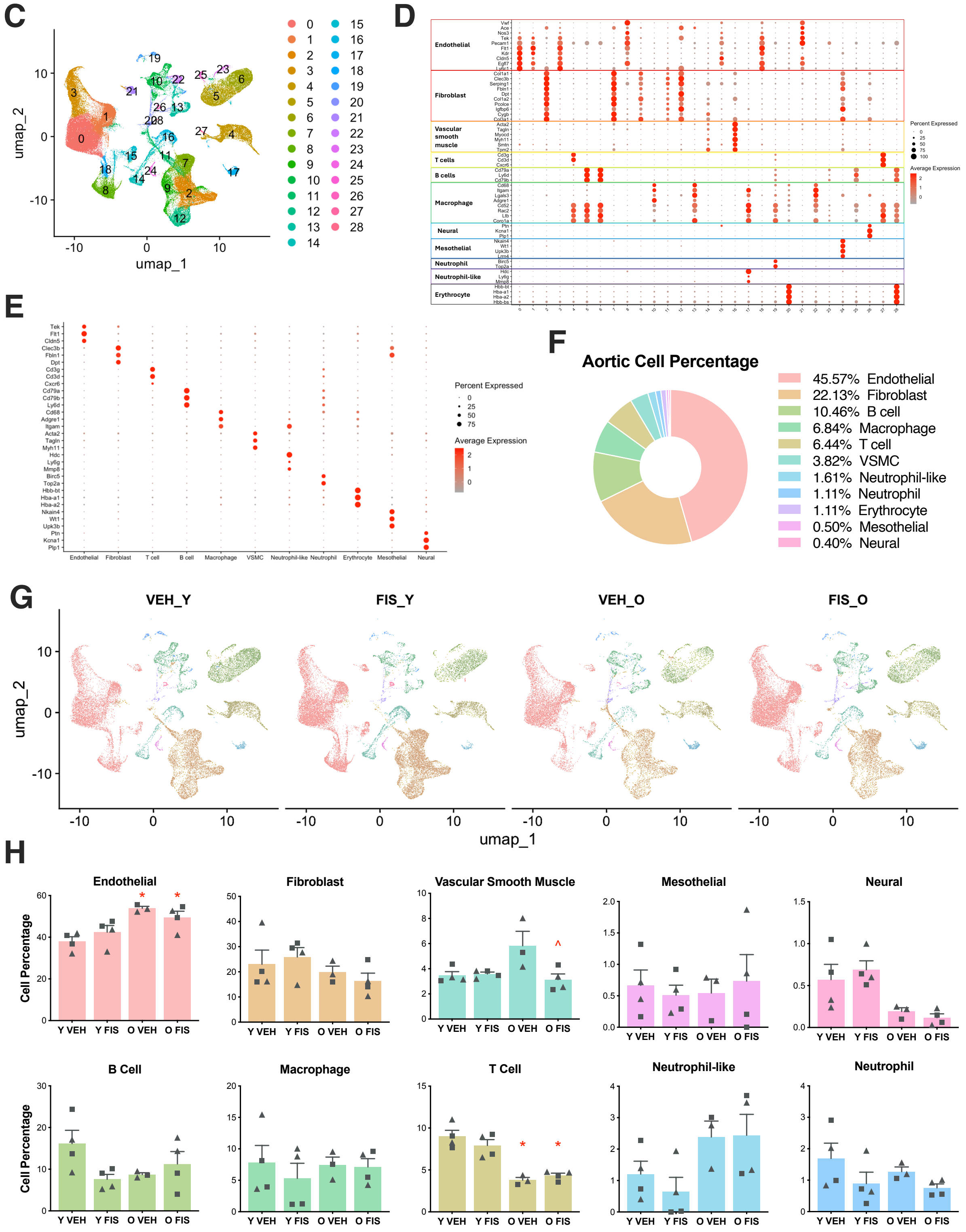

bioRxiv preprint doi: https://doi.org/10.1101/2025.08.13.670216; this version posted August 18, 2025. The copyright holder for this preprint (which was not certified by peer review) is the author/funder, who has granted bioRxiv a license to display the preprint in perpetuity. It is made available under aCC-BY 4.0 International license.

Figure S1. Whole aortic single-cell RNA sequencing quality control. Standard quality control filtering was applied to each sample to remove cells containing more than 25% mitochondrial RNAs, expressing fewer than 200 or greater than 7,000 transcripts **(A-B)**. Uniform Manifold Approximation and Projection (UMAP) of merged samples **(C)**. Cell types were identified using marker genes of each cluster **(D)**. Cell types were merged and validated by top 3 gene markers **(E)**. Aortic cell composition on merged samples by cell type **(F)**. UMAP **(G)** and cell composition analysis **(H)** of samples by cell type and treatment conditions: young vehicle (Y VEH), young fisetin (Y FIS), old vehicle (O VEH), and old fisetin (O FIS). Values represent mean ± SEM; *p<0.05 vs. Y VEH, ^p<0.05 vs. O VEH. Triangles represent females, squares represent males.

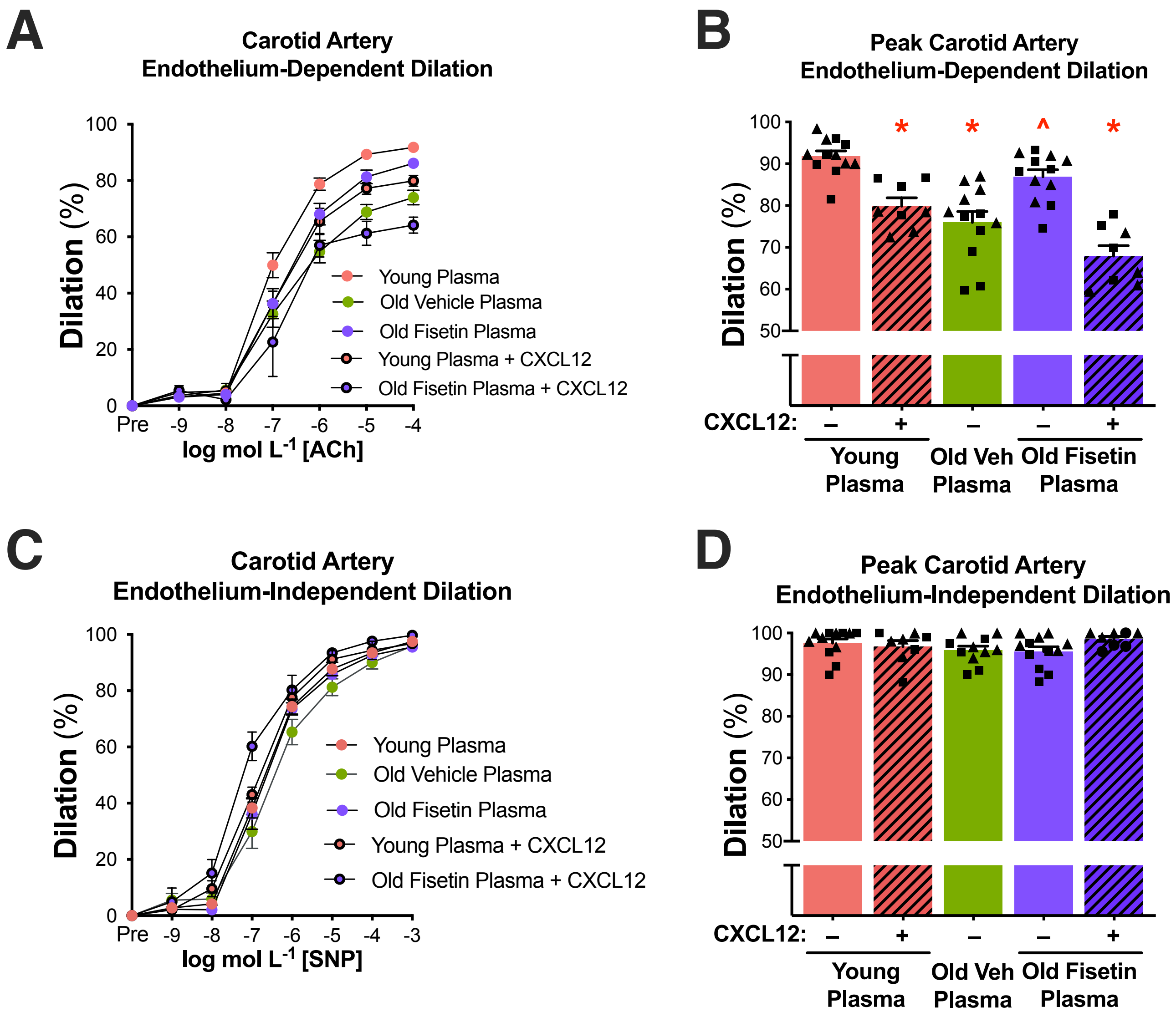

Figure S2. The circulating SASP- and CXCL12-induced endothelial dysfunction with aging and prevention by senolytic treatment with fisetin and CXCL12 inhibition. Endothelium-dependent dilation curves **(A)** and peak values **(B)** and endothelium-independent dilation curves **(c)** and peak values **(d)** in isolated carotid arteries from young mice following plasma perfusion with and without the addition of recombinant mouse CXCL12. Values represent mean ± SEM; *p<0.05 vs. young control, ^p<0.05 vs. old vehicle; triangles represent females, squares represent males. ACh: acetylcholine, SNP: sodium nitroprusside.

bioRxiv preprint doi: https://doi.org/10.1101/2025.08.13.670216; this version posted August 18, 2025. The copyright holder for this preprint (which was not certified by peer review) is the author/funder, who has granted bioRxiv a license to display the preprint in perpetuity. It is made available under aCC-BY 4.0 International license.

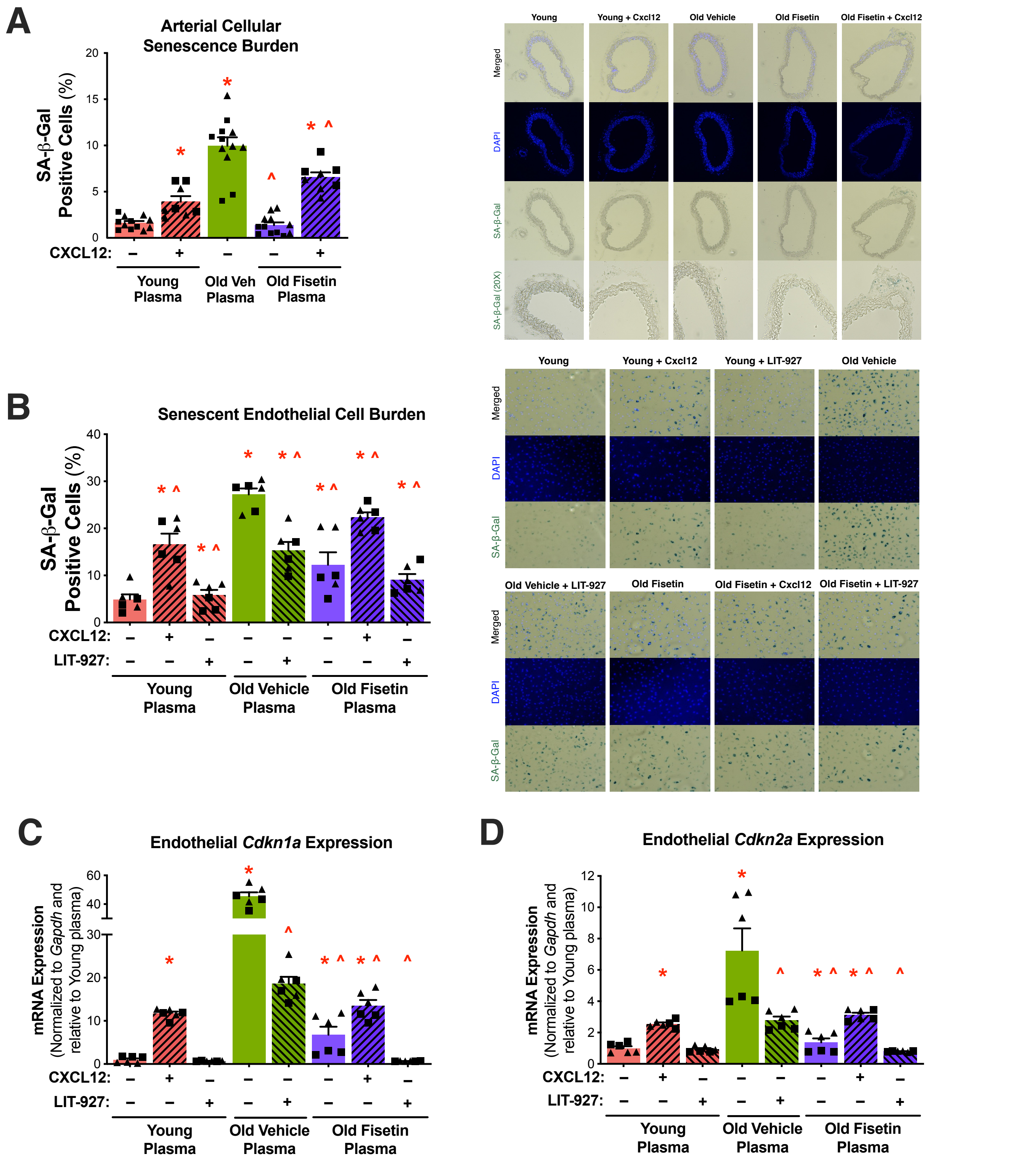

Figure S3. The circulating SASP- and CXCL12-induced cellular senescence with aging and prevention by senolytic treatment with fisetin and CXCL12 inhibition. Senescence-associated β-Galactosidase (SA-β-Gal) signal following plasma exposure with and without the addition of recombinant CXCL12 and CXCL12 inhibitor, LIT-927 in isolated arteries **(A)** and human aortic endothelial cells (HAECs) **(B)**, with representative images. Cellular senescence biomarkers *Cdkn1a* **(C)** and *Cdkn2a* **(D)** mRNA in HAECs following plasma exposure with and without the addition of recombinant CXCL12 and CXCL12 inhibitor, LIT-927. Values represent mean ± SEM; *p<0.05 vs. young control, ^p<0.05 vs. old vehicle; triangles represent females, squares represent males.

bioRxiv preprint doi: https://doi.org/10.1101/2025.08.13.670216; this version posted August 18, 2025. The copyright holder for this preprint (which was not certified by peer review) is the author/funder, who has granted bioRxiv a license to display the preprint in perpetuity. It is made available under aCC-BY 4.0 International license.

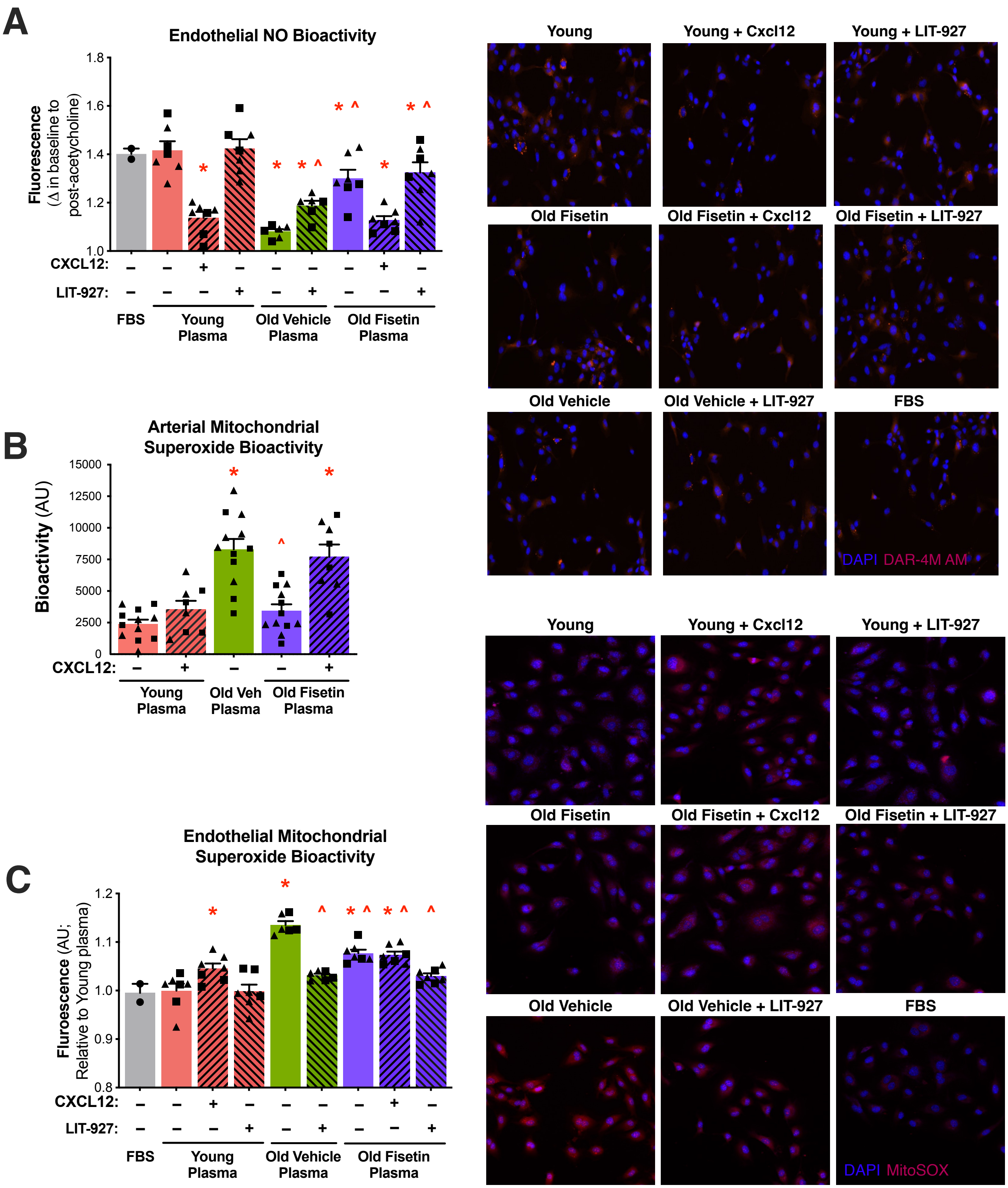

Figure S4. The circulating SASP- and CXCL12-induced acetylcholine-stimulated nitric oxide (NO) production and mitochondrial superoxide bioactivity with aging and prevention by senolytic treatment with fisetin and CXCL12 inhibition. NO bioactivity in human aortic endothelial cells (HAECs) following plasma exposure with and without the addition of recombinant CXCL12 and LIT-927 **(A)**. Mitochondrial superoxide bioactivity following plasma exposure with and without the addition of recombinant CXCL12 and LIT-927 in isolated arteries **(B)** and HAECs, with representative images **(C).** Values represent mean ± SEM; *p<0.05 vs. young control, ^p<0.05 vs. old vehicle; triangles represent females, squares represent males. FBS: fetal bovine serum, AU: arbitrary units.

bioRxiv preprint doi: https://doi.org/10.1101/2025.08.13.670216; this version posted August 18, 2025. The copyright holder for this preprint (which was not certified by peer review) is the author/funder, who has granted bioRxiv a license to display the preprint in perpetuity. It is made available under aCC-BY 4.0 International license.

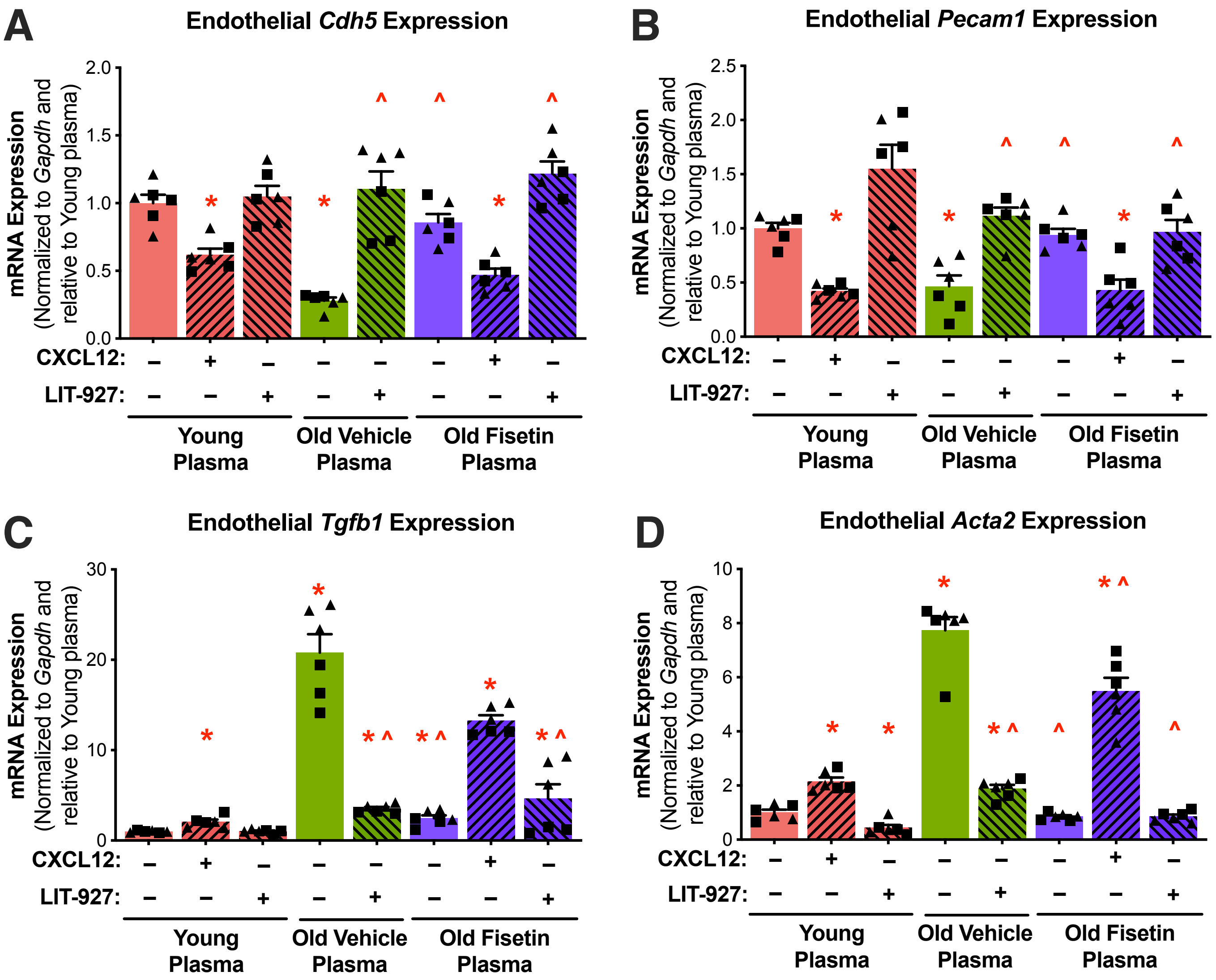

Figure S5. Fisetin supplementation prevents circulating SASP- and CXCL12-induced endothelial-to-mesenchymal transition. mRNA levels of endothelial cell biomarkers *Cdh5* **(A)** and *Pecam1* **(B)** and mesenchymal cell biomarkers *Tgfb1* **(C)** and *Acta2* **(D)** in human aortic endothelial cells (HAECs) following plasma exposure with or without the addition of recombinant CXCL12 and CXCL12 inhibitor, LIT-927. Values represent mean ± SEM; *p<0.05 vs. young control, ^p<0.05 vs. old vehicle; triangles represent females, squares represent males.
